# Current Status and Advancements in Platelet-Rich Plasma Therapy

**DOI:** 10.7759/cureus.47176

**Published:** 2023-10-17

**Authors:** Jacques Pretorius, Mohammed Habash, Bishoy Ghobrial, Rafee Alnajjar, Prasad Ellanti

**Affiliations:** 1 Trauma and Orthopaedics, University Hospital Galway, Galway, IRL; 2 Orthopaedics and Traumatology, University Hospital Galway, Galway, IRL; 3 Trauma and Orthopaedics, Letterkenny University Hospital, Letterkenny, IRL

**Keywords:** tendinopathies, osteoarthritis, narrative review, prp, musculoskeletal pathology, platelet-rich plasma

## Abstract

Platelet-rich plasma (PRP) as a treatment modality has been around for the last four decades, but only truly gained popularity over the last 10 to 15 years in medicine, in a variety of fields ranging from regenerative medicine to infertility treatment. It has gained popularity, especially in treating musculoskeletal conditions where the bulk of research has been performed and published.

There is level I evidence available supporting its efficacy in the treatment of osteoarthritis (OA), epicondylitis, bursitis, compressive neuropathy, plantar fasciitis, muscular injuries and osteochondral lesions. Most published research with regards to PRP has been focused on knee OA (limited research in shoulder, elbow, and foot and ankle OA), lateral epicondylitis and carpal tunnel syndrome, whereas spinal and hand conditions have limited research available.

Tendinopathies and partial tendon tears have conflicting evidence available, with level I evidence supporting PRP’s use in rotator cuff tendinopathies and tears, with contradictory level I evidence discouraging its use in patella and Achilles tendinopathies and tears. The available evidence regarding the use of PRP continues to produce conflicting results, but despite this, there is an ongoing increase in the popularity and use of PRP in patients with musculoskeletal conditions.

## Introduction and background

Platelet-rich plasma (PRP) as a biological agent has gained popularity over the last decade or more in treating a wide variety of conditions in different fields of medicine. PRP is derived from the centrifugation of a patient’s whole blood to produce an increased concentration of autologous platelets in a small volume of plasma [1,2]. The term was initially described in the 1970s by haematologists, who used plasma with a platelet count higher than peripheral blood as a transfusion product in thrombocytopaenia [3]. Platelets are essential for haemostasis and contain a plethora of growth factors like transforming growth factor beta-1, fibroblast growth factor, platelet-derived angiogenesis growth factor, platelet-derived growth, etc. [4]. The importance of growth factors in wound healing, chemotaxis, neovascularisation and synthesis of extracellular matrix cannot be overestimated [4]. This, in conjunction with PRP’s ability to initiate an inflammatory response through cytokine release, explains why PRP is used to augment the natural healing process and improve soft tissue healing, neovascularisation and bone regeneration [5,6].

Although PRP has been in use since the 1980s and has gained increasing popularity over time, there continue to be controversies and conflicting results regarding its effectiveness and the scope of its applicability in treating clinical conditions [7]. Today, the scope within which PRP is being used is vast, including orthopaedics, regenerative medicine (melasma, skin rejuvenation, periorbital hyperpigmentation, hair growth, scars/stretch marks, psoriasis and vitiligo), Rheumatoid arthritis, maxillofacial (temporomandibular osteoarthritis [OA]), non-diabetic foot ulcers, laryngeal application (vocal cord scarring), erectile dysfunction (Peyronie's disease), retinitis pigmentosa, vaginal atrophy and even infertility (intra-ovarian injections). In this review, the authors will focus on the newest available data regarding the use of PRP in musculoskeletal disorders.

## Review

Materials and methods

A narrative review was conducted via a comprehensive literature search in PubMed, Medline and Cochrane utilising a combination of keywords and MeSH terms. A systematic approach was used starting at the shoulders and working towards the feet, searching for all available research on all the different known musculoskeletal conditions that could be treated with injectables. The relevant data were extracted from all the identified articles to effectively describe each musculoskeletal condition that currently has research available on the use of PRP and its effectiveness. Results were limited to articles with complete results. 

The relevant data identified and discussed were arranged in this review according to the anatomical structure involved: shoulder, elbow, hand, spine, hip, knee, foot and ankle. The results of this literature review can be seen in Table 1. 

**Table 1:** Evidence available regarding the use of PRP in musculoskeletal conditions. PRP, platelet-rich plasma; GHJ, glenohumeral joint; ACJ, acromioclavicular joint; RC, rotator cuff; OA, osteoarthritis; UCL, ulnar collateral ligament; CMCJ, carpometacarpal joint; STT, scaphotrapeziotrapezoidal; TELD, transforaminal endoscopic lumbar discectomy; TFNB, transforaminal nerve root block; SIJ, sacroiliac joint; GT, greater trochanter; ACL, anterior cruciate ligament; CS, corticosteroids; HA, hyaluronic acid; N/A, not applicable; Short, short term; Mid, mid-term; Long, long term; NACD, needle aspiration of calcific deposits; IA, intra-articular; ECST, extracorporeal shockwave therapy

Anatomy	Pathology	Highest level of evidence	Efficacy in improving symptoms	Comparison to control	Follow-up period
Shoulder [9-30]	GHJ OA	Level I	Yes	> CS, = HA	Mid
	ACJ OA	Level I	Yes	N/A	Short
	RC tear	Level II	Yes	< CS Short, = CS Mid/long	Mid/long
	RC tendinopathy	Level II	Yes	> physio, > placebo	Mid/long
	Calcific tendonitis	Level I	No	NACD + PRP = NACD + placebo	Long
	Subacromial OA	Level I	Yes	= CS, = physio	Long
	Biceps tendinopathy	Level III	Yes	> CS	Short
	Frozen Shoulder	Level I	Yes	> placebo, = physio, = CS	Mid
Elbow [31-41]	Lateral epicondylitis	Level I	Yes	< CS short, > CS mid/long	Long
	Medial epicondylitis	Level I	Yes	= CS short, > CS mid	Mid
	Biceps tendinopathy	Level IV	Yes	N/A	Mid
	Triceps tendinopathy	Level V	Yes	N/A	Mid
	UCL injury	Level IV	Yes	N/A	Mid
	Carpal Tunnel	Level I	Yes	> CS, splinting and placebo	Mid
Hand [42-49]	De Quervains	Level I	Yes	< CS short, >CS mid	Mid
	CMCJ OA	Level I	Yes	= CS short,= CS mid, > CS long	Long
	STT OA	Level IV	No	N/A	Mid
	Trigger finger	Level V	Yes	N/A	Mid
Spine [50-58]	Disc pathology	Level II	Yes	= CS	Mid
	TELD with PRP	Level II	Yes	> TELD alone	Long
	TFNB	Level I	Yes	= CS	Long
	Facet Joint OA	Level I	Yes	> CS	Mid
	SIJ OA	Level II	Yes	= PRFibrin, =CS	Mid
Hip [59-64]	Hip OA	Level I	Yes	= CS, = HA	Long
	GT bursitis	Level I	Yes	=/> CS	Mid/long
Knee [65-92]	Knee OA	Level I	Yes	> CS mid/long	Mid/long
	Intraosseus	Level I	No	= IA PRP alone	Mid
	Meniscus (repair+PRP)	Level I	Yes	> repair alone	Mid
	Meniscus PRP	Level IV	Yes	N/A	Mid
	ACL (recon/PRP)	Level I	No	= ACL recon alone	Mid
	Patella tendinitis	Level I	No	= HA, CS, Placebo, stem cells, high volume saline	Long
	Muscles	Level II	Yes	> physio	Mid
	Osteochondral	Level I	Yes	> microfracture alone	Mid
	Wound healing	Level II	Yes	> placebo	Short
Foot and ankle [93-110]	Foot and ankle OA	Level I	No	= placebo	Mid
	Osteochondral	Level I	Yes	> surgery alone, > HA	Mid
	Plantar fasciitis	Level I	Yes	> CS, > ECST, = autologous blood	Mid
	Achilles tendinopathy	Level I	No	= placebo = debridement alone	Mid
	Achilles tear	Level II	No	= rest, = physio	Mid
Fractures [111,112]	Non-union	Level IV	No	N/A	Mid
	Non-union	Level I	Yes	Revision surgery + PRP > Surgery + placebo	Long

All included studies were classified, by two independent reviewers, according to the level of evidence as described by the Oxford Centre for Evidence-Based Medicine [8]. The authors simplified the classification by not discussing the sub-classifications and only classifying a study as level I, II, III, IV or V. There was no specific reference to a case report, and therefore, this was added as level V in the classification system (Table 2).

**Table 2:** Levels of evidence for therapeutic studies. RCT, randomised controlled trial

Level	Type of evidence
I	Systematic review (with homogeneity) of RCTs; Individual RCT (with narrow confidence intervals)
II	Systematic review (with homogeneity) of cohort studies; Individual Cohort study (including low-quality RCTs, e.g., <80% follow-up), *Outcomes* research; Ecological studies
III	Systematic review (with homogeneity) of case-control studies; Individual Case-Control study
IV	Case series (and poor-quality cohort and case-control study)
V	Expert opinion without explicit critical appraisal or based on physiology bench research or *first principles*, case study

Shoulder

Glenohumeral and sub-acromial PRP injections are indicated for a wide variety of shoulder-related soft-tissue and bony conditions. First, looking at the evidence supporting its use in OA of the shoulder joint, in contrast to knee OA, the number of studies available for glenohumeral OA is limited with only two randomised controlled trials (RCTs). PRP was shown to be an effective treatment in improving pain and function in both studies [9,10], with superiority to corticosteroids, especially at long-term follow-up [9] and no difference when compared with hyaluronic acid [10].

Furthermore, to the best of the authors' knowledge, no RCT was available investigating the role of injectables in the treatment of Acromioclavicular joint OA, although three prospective studies demonstrated symptom improvement after corticosteroid injections [11-13]. Only one non-comparative prospective pilot study with a small population size demonstrated promising results for PRP injections into the ACJ with statistically significant clinical improvement in symptoms over the short term and mid-term [14].

Rotator cuff injuries can lead to significant morbidity, especially in elderly patients, and surgery might not always be the best solution, with physiotherapy and injectables available as a successful treatment option in partial rotator cuff tears or tendinopathy. A systematic review (SR) and meta-analysis, consisting of six RCTs, it was demonstrated that PRP and corticosteroid injections produced similar outcomes in terms of pain relief and functional improvement over medium- to long-term follow-up, with corticosteroids delivering better results during the first six weeks [15]. A well-designed RCT involving 99 patients with partial-thickness rotator cuff tear or rotator cuff tendinopathy revealed that, at three months, PRP showed superiority over corticosteroids in terms of pain and function. However, no significant difference was observed at one year [16]. 

A meta-analysis assessing PRP treatment in rotator cuff tendinopathy compared its outcomes with physiotherapy alone, placebo injections or no treatment. The analysis revealed superiority in pain improvement over the medium to long term, while no significant difference was observed in terms of function [17].

Rotator cuff calcific tendonitis is commonly treated with needle aspiration of calcific deposits (NACD) and corticosteroid injection. This was compared to PRP post-NACD in an RCT of 80 patients, which demonstrated worse clinical scores at six weeks but better clinical scores for PRP at six months with no difference at one to two years follow-up [18]. However, when compared to placebo, there was no statistically significant difference, indicating that NACD is most likely the major predictor of outcome [19].

It has been proven that PRP injections are an effective treatment in improving pain and function in patients with subacromial impingement (SAI) [20]. However, when comparing its effectiveness to corticosteroid injections, there were contradictory results. As demonstrated in an RCT performed by Pasin et al. [21] who included 99 patients with confirmed SAI, these patients were divided into three groups: PRP, corticosteroids and physiotherapy, with the PRP group demonstrating superior scores at eight weeks with all assessed outcomes (Quick Disabilities of the Arm, Shoulder, and Hand [QuickDASH], the University of California, Los Angeles (UCLA) Shoulder Rating Scale and the Visual Analogue Scale [VAS]). However, the RCT performed by Baretto et al. [22] demonstrated no significant difference at all time points (six weeks, three months and six months) when comparing PRP with corticosteroids in a cohort of 51 patients. Another RCT even demonstrated no statistically significant difference between PRP and physical therapy alone [23].

The long head of the bicep’s tendinopathy would generally be considered a surgical condition, but available evidence demonstrates that injectables have an important role to play in managing this condition [24-26]. This injection therapy has a favourable outcome when performed under ultrasound (U/S) guidance [24]. Only one case-control study is available assessing the role of PRP, which demonstrated that PRP was superior to corticosteroids in pain improvement and function [25].

Adhesive capsulitis (AC) is a common debilitating shoulder condition, which is generally treated with corticosteroid injections (demonstrated to be more effective than HA) and physiotherapy with high success rates [27]. With the increasing popularity of PRP, its efficacy has been compared with different control groups in two RCTs: shown to be as effective as physiotherapy, which could be useful in patients less prone to follow an exercise program [28] and superior to placebo with regards to pain, disability and ROM [29]. The study performed by Thu et al. [28] divided 64 patients into two equal groups, with one receiving PRP injections and the other receiving an intensive physiotherapy program of three sessions weekly for six weeks. Both groups had reduced VAS and shoulder and hand scores with improved ROM, and there was no statistically significant difference between the two groups. A cohort study showed improvement in pain, movement and function comparable to corticosteroid injections for AC [30]. 

Elbow

Abundant data are available for PRP injections in patients with lateral epicondylitis. Multiple SRs offer contradictory results, which causes an ongoing controversy. One review, including 31 trials comparing different injectables, concluded that corticosteroids and botulinum toxin resulted in an improved clinical outcome in the short term, whereas PRP and autologous blood injections did not provide any statistically significant benefit over placebo [31]. However, another SR identified PRP to be superior to corticosteroids and autologous whole blood in terms of pain and function in the long term [32]. In an SR of five other SRs looking at the difference between corticosteroid and PRP injections, it was concluded that corticosteroids were more effective in the short term, with PRP being a more effective long-term treatment option [33].

In contrast to lateral epicondylitis, there is a paucity of studies available assessing the use of PRP in the Golfer’s elbow (medial epicondylitis). One RCT including 83 patients with both medial (*n *= 20) and lateral epicondylitis (*n *= 63) demonstrated superior outcomes of PRP at six months, but no distinction was made between the two conditions in the results and discussion section [34]. 

Only two small prospective non-comparative studies showed that U/S-guided PRP injections are an effective treatment method for patients affected by distal biceps tendonitis, which was confirmed on MRI imaging [35,36]. The paucity of research available for distal triceps tendinopathy is even more obvious, with only one case report available, which supports the effectiveness of its use, in conjunction with a rehabilitation program [37].

Overhead athletes with ulnar collateral ligament (UCL) elbow injuries can be successfully managed conservatively, with PRP injections, as supported by three separate case series [38-40]. A prospective cohort study, including 50 patients, was recently performed, which showed a high rate of success with PRP injection in conjunction with a rehab programme for patients with grades I, II and III UCL injuries with a high rate of return to play (RTP) [41].

To the authors' knowledge, there are no studies available assessing the use of PRP injections in patients with ulnohumeral and radiocapitellar OA. This is likely due to the low incidence of symptomatic elbow OA, and therefore, the need for injectable therapies is low, which includes PRP.

Hand

Carpal tunnel syndrome is the most common compressive neuropathy, and as would be expected, it has an abundance of research available, including research assessing the use of PRP. In a recent SR and meta-analysis comprising eight RCTs, PRP injections were compared to corticosteroids, placebo or wrist splinting. It was found that PRP is more effective than other conservative modalities with regards to relieving pain, improving wrist function and partially improving electrophysiological indicators up to mid-term follow-up [42]. There is still a need to investigate and clarify the long-term effects of PRP with RCTs with longer study periods. Carpal tunnel syndrome is the only compressive neuropathy that has been thoroughly assessed for the effectiveness of PRP, whereas cubital tunnel and tarsal tunnel syndromes are yet to be investigated.

De Quervain’s tenosynovitis is frequently managed with corticosteroid injections with evidence supporting its use, but limited studies exist on the role of PRP. In a trial performed on 40 patients, the corticosteroid group produced better pain relief, improved hand function and U/S findings in the short term, but PRP was found to be statistically superior to corticosteroids at mid-term follow-up. The authors believed this to be due to PRP’s ability to stimulate the body’s regenerative abilities [43]. This finding was also supported by a cohort study demonstrating the benefit of PRP injection in De Quervain's tenosynovitis [44].

Carpometacarpal joint (CMCJ) OA is the second most common OA in the hand. Despite this, only a few studies have investigated the use of PRP in this condition. One RCT exists, which demonstrated a superior response of PRP to corticosteroids at 12 months follow-up with regards to pain, function and patient satisfaction [45]. A small prospective cohort study produced similar results at six months [46]. In contrast to this, a small retrospective analysis of 28 patients receiving PRP injections for either CMCJ or scaphotrapeziotrapezoidal (STT) joint OA did not show any obvious improvement [47].

According to the authors' knowledge, only one case report exists assessing the use of PRP in patients with a trigger finger, which produced an excellent response with complete recovery at 12 weeks and no further triggering or discomfort thereafter [48]. Currently, there is an ongoing RCT comparing PRP with corticosteroid and placebo injections, which will hopefully provide more concrete evidence supporting PRP as a possible alternative treatment option [49].

Spine

Lower back pain (LBP) has different modalities of treatment, and there has been some interest in whether PRP might be an efficient modality in treating specific conditions causing LBP. There are relatively few studies available supporting its use in spinal conditions. An SR identified that intra-discal PRP injections may be an effective treatment for discogenic back pain. This included one RCT that reported positive results, but the study had major methodological flaws. The overall success rate for PRP in aggregate was 54.8% after assessing 12 included studies, where an outcome was determined to be successful if it relieved more than 50% of the patient's symptoms post-therapy [50].

A large prospective cohort study showed promising results for transforaminal endoscopic lumbar discectomy (TELD) in combination with PRP injection when compared to isolated TELD. The combination therapy produced improved clinical symptoms, improved remodelling and decreased reoccurrence rates in the mid- to long-term follow-up [51]. 

Similarly, a large RCT demonstrated that transforaminal injections with PRP for patients with radicular pain due to lumbar disc herniation significantly improved patients’ symptoms for up to one year, which was comparable to steroid injection [52]. Facet joint injections were also supported by three level IV studies, as summarised by Desai et al. [53] and one RCT, which showed superior results at six months when compared to corticosteroid injections [54].

Sacro-iliac joint pathology is an important differential in patients affected by LBP. A recent descriptive review identified a total of seven studies available assessing the use of PRP in these patients. This literature suggests that PRP has favourable pain and functional primary outcomes with no major adverse events [55]. The highest quality study that exists is a non-RCT, which demonstrated similar outcomes when compared to platelet-rich fibrin injections [56] and another comparative study, which demonstrated similar results to corticosteroid injections [57]. Platelet-rich fibrin is a second-generation platelet concentrate consisting of an immune and platelet concentrate collected on a fibrin membrane [58].

Hip

Hip OA treated with intra-articular PRP injections does not have nearly the same bulk of literature available as knee OA, but the reported outcomes are similar. Multiple studies demonstrated that U/S-guided intra-articular PRP injections are an effective treatment in improving symptoms for up to 12 months, with the most significant improvement at four months [59-61]. There has been no statistically significant difference in outcome demonstrated between HA and PRP injections in a meta-analysis performed by Berney et al. [60]. Another meta-analysis performed, which included 11 RCTs, showed that all intra-articular injections (PRP, corticosteroids, HA and placebo) demonstrated statistically significant clinical and functional improvement, but interestingly there was no significant difference when compared to placebo [59].

The role of PRP in the treatment of greater trochanteric (GT) bursitis or gluteal tendinopathy is still controversial, with four level II studies available producing conflicting results. Two RCTs demonstrated significant improvement in symptoms over corticosteroids at mid- to long-term follow-up [49,62]; another prospective comparative study only assessed short-term follow-up and showed no improvement in symptoms for PRP, whereas steroid injections produced significant improvement [63]. One RCT even demonstrated no difference in pain and functional outcome between placebo and PRP injection for patients with GT bursitis up to one year [64].

Knee

PRP injections of the knee for OA have been widely investigated with an overly positive outcome with significant improvement in symptoms. A meta-analysis, which included 30 RCTs with a total of 3,463 patients, concluded that PRP had the best overall outcome at 12 months in comparison to corticosteroids, hyaluronic acid and placebo [65], and improvement has even been noted up to five years [66]. It has been proven to be statistically significant that PRP does improve patients’ symptoms with mild-to-moderate knee OA about pain, stiffness and function. The most significant benefit of PRP over the other injectable therapies is the longer lasting effects, with a low risk of adverse events [66-68]. The use of single versus multiple PRP injections has been investigated at different time intervals with conflicting results either indicating no significant difference [69,70] or significant improvements when multiple injections were used as demonstrated in an RCT with 162 participants [71]. 

Following on the effectiveness of intra-articular PRP injections, there has recently been a movement to use intra-osseous injections as they might more effectively treat and restore the subchondral bony structure. The data available are still limited, with only one RCT available comparing intra-articular PRP injection with or without intra-osseous injections, which showed no additional benefit over only intra-articular injections for mid-term follow-up [72]. The results of multiple prospective studies performed were uniform in supporting the use of PRP intra-articular with intra-osseous PRP injections with regards to pain relief and functional improvement when compared to only PRP intra-articular injection [73], hyaluronic acid [74] and no comparison [75,76].

Meniscal lesions can be considered for conservative management if it is of a stable traumatic or degenerative origin. Unstable and symptomatic meniscus injuries would generally be managed arthroscopically with debridement or repair, but the role of adjuvant PRP injections has been under investigation. An SR performed suggested that PRP can effectively enhance arthroscopic repair with reduced failure rates, decreased severity of pain and improved range of motion [77]. This was contrasted by a recent RCT that suggested that adjuvant PRP resulted in similar functional outcomes and healing rates [78]. PRP has been suggested to be effective in improving symptoms for patients with stable traumatic meniscus injuries treated conservatively [79,80], as well as degenerative meniscal lesions [81].

Many RCTs are available to determine the benefit of PRP injections with ACL reconstruction, which was analysed with an SR, which demonstrated no significant difference with regards to pain, function, anterior tibial translation, tunnel widening after graft fixation as well and graft integration [82].

There was no statistically significant difference identified between PRP and other injectable therapies (hyaluronic acid, stem cells, high-volume image-guided saline and corticosteroid, dry needling, and placebo) for patella tendinopathy in an SR and meta-analysis performed [83]. The study did demonstrate significant benefit in favour of PRP in comparison to extracorporeal shock wave therapy.

Patients affected by muscle injuries would frequently entail young active individuals with a desire to return to sport as judiciously as possible. PRP injections have been used for a wide variety of muscular injuries, with the majority of the studies centred on hamstring injuries. An SR and meta-analysis reviewed 10 studies and found that PRP injections reduced the time to RTP as well as reinjury rates when compared to no treatment or physiotherapy alone, although this was not statistically significant [84]. Trunz et al. and Bradley et al. published cohort studies, which demonstrated statistically significant improvement in RTP after haematoma aspiration and PRP injections [85,86], with an improved outcome in managing acute rather than chronic injuries [87]. Studies examining other quadriceps and gastrocnemius muscle injuries have reported similar results [82]. Another potentially underdiagnosed sporting injury is piriformis syndrome, which showed superior results when treated with PRP injections compared to placebo in an RCT [88].

With regards to the use of PRP in osteochondral lesions in the knee, multiple RCTs have been published that expressed the superiority of microfracture with PRP injection with regards to functional recovery and resolution of pain [89,90], even demonstrating improved symptomatology up to two years [91]. A recent pilot study illustrated promising results for both PRP and adipose-derived mesenchymal stem cells in conjunction with microfracture, which seems to enhance the cartilage restoration ability [92].

Trams et al. also systematically reviewed six RCTs that assessed the benefit of PRP in reducing blood loss during and after TKA surgery, which demonstrated a lower calculated blood loss and a lower haemoglobin drop after surgery [82]. 

Foot and ankle

When assessing tibiotalar pathology, the two most important conditions to consider for possible PRP injections would include OA and osteochondral lesions. In contrast to the abundance of studies available for PRP in knee OA, only one RCT, according to the authors' knowledge, has been published, including 100 participants, which showed that there was no statistically significant difference between PRP and placebo injections [93]. This was contrasted by two other prospective non-comparative studies [94,95], as well as two retrospective studies, which suggest that PRP may be a safe and effective alternative to postponing surgical management [96,97].

An SR performed on the use of PRP in osteochondral lesions found that PRP in conjunction with microfracture produced superior results about function and pain relief in small lesions when compared to surgery alone, which was supported by a recent RCT performed [98,99]. It also highlighted that PRP was more effective in relieving symptoms than hyaluronic acid [99]. 

In the context of plantar fasciitis, a substantial body of evidence is available. Two recent systematic reviews suggest that PRP is more effective than corticosteroid injections in addressing pain and improving function during mid-term follow-up [100,101]. Hohmann et al. highlighted that, of the 15 studies, there were multiple studies with low quality, nine with a high risk of bias and different protocols for PRP preparation were used. All these factors could decrease the internal and external validity of these findings [100]. A recent large high-quality RCT including 118 patients showed increased benefit of PRP over steroid injection at six months follow-up [102]. Comparison with other conservative treatment options is limited, but one large RCT demonstrated PRP’s superiority to extracorporeal shockwave therapy with regards to pain alleviation [103], whereas another RCT did not demonstrate any statistically significant difference between PRP and autologous blood injection [104]. Interestingly, one RCT used MRI imaging to predict if any specific features might influence the outcome of PRP and corticosteroid injections, which showed that steroid injections were only effective if the initial fascia thickness was >7 cm, whereas PRP was effective regardless of thickness [105].

The use of PRP in Achilles tendinopathy and acute tendon rupture is inefficient, with an SR and meta-analysis of four RCTs demonstrating no clear benefit of PRP in managing chronic mid-substance tendinopathy in comparison to placebo [106]. It was also demonstrated by another meta-analysis assessing the use of PRP in acute Achilles tendon ruptures that there is no evidence supporting an increased efficacy with regards to the plantar flexion angle, strength as well as pain [107] and supported by a recent RCT [108]. Endoscopic debridement of chronic non-insertional Achilles tendinopathy with PRP injections demonstrated no benefit over debridement alone [109].

A small case series of hallux sesamoid injuries treated with a single PRP injection demonstrated an increased return to impact sport rate, which could be an interesting field to consider for further higher-quality studies [110].

Non-union

The use of PRP in non-union is an exciting prospect as this is a condition with high morbidity and significant financial implications. As noted by Andersen et al., the consensus currently in literature is that PRP is an effective alternative (or adjunct) treatment option for patients with non-union fractures [111], although it should be noted that there are no recent high-quality studies available assessing the use of PRP in isolation for non-union fractures. However, PRP in conjunction with revision surgery and bone grafting has a well-designed RCT showing promising results with higher cure rates, shorter healing duration and better pain relief when compared to placebo injection [112]. 

## Conclusions

This is an exciting era for regenerative musculoskeletal medicine with PRP as the forerunner, as our understanding and utilization of PRP continue to advance. PRP is a promising conservative treatment modality for a variety of conditions with healing and regenerative capabilities and clear evidence of safety. The heterogeneity of PRP preparations, outcome measures and study designs has made interpreting current literature difficult and limits our ability to make treatment recommendations. This study attempted to use the available literature to summarise the following recommendations.

Level I research supporting the use of PRP in OA is available, with a substantial body of evidence supporting its effectiveness in knee and hip OA. However, there is limited research on its application in spine, hand, and shoulder OA, and no studies supporting its use in elbow and ankle OA. High-volume level I research supports its use in lateral epicondylitis, trochanteric bursitis, plantar fasciitis and carpal tunnel neuropathies. Tendinopathies and partial tendon tears have conflicting evidence available, with level I evidence supporting PRP’s use in rotator cuff tendinopathies and tears, with contradictory level I evidence discouraging its use in the patella and Achilles tendinopathies and tears. There are also conflicting results when PRP is used in conjunction with reconstruction surgery, with its addition providing superior outcomes in patients undergoing meniscus repair, osteochondral microfracture, and revision surgery for non-union. However, no improvement is observed over isolated ACL reconstruction. Limited studies support its potential as a conservative treatment modality for conditions like trigger finger, collateral ligament injuries, calcific tendonitis and frozen shoulder. Despite ongoing conflicting results, there is a continuous increase in the popularity and use of PRP, which is undeniable.
